# Novel biallelic variants in IREB2 cause an early-onset neurodegenerative disorder in a Chinese pedigree

**DOI:** 10.1186/s13023-024-03465-7

**Published:** 2024-11-25

**Authors:** Zhenglong Guo, Dawei Huo, Yingying Shao, Wenke Yang, Jinming Wang, Yuwei Zhang, Hai Xiao, Bingtao Hao, Shixiu Liao

**Affiliations:** 1 Henan Provincial Key Laboratory of Genetic Diseases and Functional Genomics, Medical Genetics Institute of Henan Province, Henan Provincial People’s Hospital, People’s Hospital of Zhengzhou University, Zhengzhou, 450000 China; 2 School of Medicine, People’s Hospital of Henan University, Henan University, Zhengzhou, 450000 China; 3 Bone Marrow Transplantation Center of The First Affiliated Hospital & Liangzhu Laboratory, Institute of Hematology, Zhejiang Province Engineering Research Center for Stem Cell and Immunity Therapy, Zhejiang University School of Medicine, Zhejiang University, Hangzhou, 310003 China; 4 The First Affiliated Hospital of Zhengzhou University School, Zhengzhou, 450000 China; 5 Department of Immunology, School of Basic Medical Sciences, Zhengzhou University, Zhengzhou, 450001 China; 6 Henan Eye Institute, Henan Academy of Innovations in Medical Science, Zhengzhou, Henan 450000 China

**Keywords:** IREB2, Biallelic variants, Early-onset neurodegeneration, Iron metabolism homeostasis

## Abstract

**Background:**

Cellular iron metabolism is essential for maintaining various biological processes in organisms, and this is influenced by the function of iron-responsive element-binding protein 2 (IRP2), encoded by the *IREB2* gene. Since 2019, three cases of a genetic neurodegenerative syndrome resulting from compound heterozygous mutations in *IREB2* have been documented, highlighting the crucial role of IRP2 in regulating iron metabolism homeostasis. This study aims to investigate the molecular basis in a single proband born to non-consanguineous healthy parents, presenting with severe psychomotor developmental abnormalities and microcytic anemia.

**Methods:**

Trio-whole exome sequencing (WES) was applied to identify the disease-causing gene in an 8-month-old male patient from China. In silico tools were used to predict the pathogenicity of the identified variants, and in vitro functional studies were performed to evaluate the molecular mechanism.

**Results:**

WES identified novel biallelic variants, c.1111 A > G (P.Ile371Val) and c.2477 A > T (P.Asp826Val), in the *IREB2* gene, which encodes the iron metabolism-related protein, IRP2. Functional studies revealed that c.2477 A > T resulted in a significant degradation of IRP2, which led to the misregulation of intracellular ferric iron.

**Conclusions:**

We report the identification of the first functional domain associated with the degradation of IRP2. The biallelic variants that affect protein degradation likely underlie the pathogenesis of the IRP2-related neurodegenerative disorder. Moreover, the use of proteasome inhibitors can potentially restore the expression of IRP2, highlighting a promising therapeutic target for patients with IRP2deficiency.

**Supplementary Information:**

The online version contains supplementary material available at 10.1186/s13023-024-03465-7.

## Background

Iron is essential for the growth and replication of mammalian cells. It acts as a cofactor in forming heme and iron-sulfur clusters (Fe-S), which are crucial for hemoglobin synthesis, mitochondrial function, inflammatory response, and lipid metabolism [1, 2]. Disruption in iron metabolism homeostasis can lead to various human diseases including anemia [3], aging [4], cardiovascular disease [5, 6], and neurodegenerative conditions such as Parkinson’s and Alzheimer’s disease [7–9].

Intracellular iron metabolism homeostasis relies on two homologous cytosolic iron regulatory proteins, IRP1 and IRP2. These proteins interact with iron-responsive element (IRE) in mRNA to post-transcriptionally regulate the expression of genes related to iron metabolism, such as heavy and light chain ferritin (FTH and FTL) and transferrin receptor (TFRC) [10, 11]. In iron-deficient cells, IRPs bind to the IRE in the 5’-UTR of FTH and FTL mRNA, inhibiting their translation and thereby reducing iron storage and excretion. Simultaneously, they bind to the IRE in the 3’UTR of TFRC mRNA, enhancing its stability to promote iron absorption and utilization. Conversely, under iron-replete conditions, IRP1 acquires aconitase activity by binding to Fe-S, becoming holo-IRP1 and losing its IRE binding ability. IRP2, lacking an iron-sulfur cluster and aconitase activity, undergoes degradation via FBXL5-mediated ubiquitination, promoting iron storage and excretion, while inhibiting iron absorption [12–14]. Studies in transgenic mice have demonstrated that the deletion of IRP1 impacts iron metabolism only in certain tissues such as kidneys and adipose [15]. In contrast, IRP2 compensates for the loss of IRP1, and its knockout affects iron metabolism in systemic tissues. This deficiency leads to progressive adult-onset neurodegenerative symptoms, including ataxia, bradykinesia, and tremor, after six months of age [16, 17]. These findings suggest a link between the loss of IRP2 function and the misregulation of iron metabolism, contributing to the pathological progression of neurodegenerative diseases.

In 2019, the first case of adult-onset neurodegeneration caused by compound heterozygous nonsense mutations in *IREB2* was reported [18]. Since then, three patients have been identified through whole exome sequencing (WES), all exhibiting neurological and hematological symptoms similar to those observed in IRP2 knockout mice [18–20]. This condition, termed Neurodegeneration, Early-onset, with Choreoathetoid Movements and Microcytic Anemia (NDCAMA, OMIM#618451), highlights the critical role of IRP2 in neurological health. Mechanistic studies have revealed that IRP2 plays a key role in mitochondrial function by stabilizing hypoxia-inducible factors, thereby regulating the metabolic switch from glycolysis to oxidative phosphorylation [21]. Notably, cells derived from the third NDCAMA patient, who harbored missense mutations, showed significant degradation of the IRP2 protein. This degradation is possibly due to abnormal mRNA splicing, although conclusive evidence is still lacking [20]. This growing body of evidence underscores the importance of IRP2 in maintaining iron metabolism and mitochondrial function, and its disruption leads to severe neurological and hematological manifestations.

Here, we describe a new NDCAMA patient with novel biallelic missense variants in *IREB2*, manifested as developmental delay, epilepsy, hypertonia, and microcytic anemia. This may be attributed to IRP2 degradation-induced iron metabolism abnormalities. These findings suggest a potential link between missense mutations and IRP2 degradation in the pathogenesis of NDCAMA.

## Materials and methods

### Cell culture

SH-SY5Y cell line was obtained from the American Type Culture Collection (ATCC) (CA, USA) and cultured in Dulbecco’s Modified Eagle’s Medium (DMEM, Gibco, USA) containing 10% fetal bovine serum (FBS) and 1% streptomycin/penicillin. These cells were maintained in constant-temperature incubator at 37 °C with 5% CO2. To inhibit IRP2 protein degradation, cells were treated with 40 μM MG-132 (AbMole, USA) for 8 h, and cells treated with an equal volume of DMSO served as control group. Total protein was then extracted for western blot analysis.

### Real-time qPCR

Peripheral blood mononuclear cells (PBMCs) were isolated from patient and an age-matched healthy control. Total RNA was extracted using TRIzol (Invitrogen, CA, USA) according to the manufacturer’s instructions. Then the purity and concentration of the RNA was determined by the Nanodrop (Thermo, MA, USA). cDNA was generated using RevertAid RT kit (Thermo, MA, USA). qPCR was performed using Taq Pro Universal SYBR qPCR Master Mix (Vazyme, Nanjing, China). Primer sequences are shown in Supplemental Table S1. Quantification of data was performed using 2 − ΔΔCt method and ACTB was used as housekeeping gene for normalization.

### Western blot

Total protein of cells from different group was extracted using RIPA lysis buffer containing 1mM PMSF (Solarbio, Beijing, China) and the concentration was measured using BCA Protein Quantification Kit (Vazyme, Nanjing, China). Gel electrophoresis, transmembrane and protein detection was performed as described [22]. The following antibodies were used: anti-IRP2 antibody (1:500 dilution; SantaCruz, sc33682), anti-FTH antibody (1:1000 dilution; Affinity, DF6278), anti–TFRC antibody (1:1000 dilution; Proteintech, 10084-2-AP), anti-Flag antibody (1:5000 dilution; HUABIO, M1403-2), anti–GFP antibody (1:50000 dilution; Proteintech, 66002-1-Ig), anti–ACTB antibody (1:3000 dilution; Proteintech, 66009-1-Ig), HRP-conjugated Goat Anti-Rabbit antibody (1:10000 dilution; Proteintech, SA00001-2) and HRP-conjugated Goat Anti-Mouse antibody (1:10000 dilution; Proteintech, SA00001-1). Relative protein expression was analyzed using Image J software.

### Overexpression of wild-type and mutant IRP2

Human IREB2 coding sequence (Gene ID: 3658) was used to construct the Flag-*IREB2*-WT, Flag-*IREB2*-A1111G and Flag-*IREB2*-A2477T plasmids, followed by lentivirus packaging and validation (OBIO Biosciences, Shanghai, China). Then the SH-SY5Y cells were infected with corresponding IRP2-overexpression lentivirus, followed by 2 µg/mL puromycin (Solarbio, Beijing, China) selection to obtain stable express cells, while the backbone lentivirus infected cells were used as control group.

### Intracellular Fe^2+^ detection

FerroOrange fluorescent probe (Dojindo, Japan) was applied to detect intracellular Fe^2+^ according to the manufacturer’s protocol. In brief, 1 × 10^5^ SH-SY5Y cells were seeded in 96-well plates and cultured for 24 h. Subsequently, the cells were rinsed with PBS and incubated with 1 µM FerroOrange diluted in serum-free medium for 30 min. 540 nm excitation and 585 nm emission was performed to measure the absorbance using a microplate reader (BioTek Instruments, USA).

### In-silico predictions

In silico analyses was performed to annotate the pathogenicity of missense variants using various online softwares including SIFT (https://sift.bii.a-star.edu.sg/), Polyphen-2 (http://genetics.bwh.harvard.edu/pph2/), VarSite (https://www.ebi.ac.uk/thornton-srv/databases/VarSite), MutationTaster (https://www.mutationtaster.org/), Missense3D (http://missense3d.bc.ic.ac.uk/missense3d/) and DUET (https://biosig.lab.uq.edu.au/duet/stability). Amino Acid Sequences of IRP2 from different mammalian species were downloaded from UniProt database (https://www.uniprot.org/), followed by sequence alignment using the WebLogo tool (https://weblogo.threeplusone.com/create.cgi). AlphaFold v3 (https://golgi.sandbox.google.com) was used to establish the stucture of IRP2. The three-dimensional (3D) structure of the interactions between wild-type and mutated IRP2 protein and targeted protein were obtained from Protein Data Bank (https://www.rcsb.org) and was examined using Pymol software (https://www.pymol.org).

### Statistical analysis

Data were presented as means ± SD, and two-tailed Student’s *t*-test was used to test for statistical differences between two groups. For more groups, one-way ANOVA with Dunnett’s multiple comparison test was performed. P value of < 0.05 was considered statistically significant.

## Results

### Clinical characterization of the patient

**Fig. 1:**
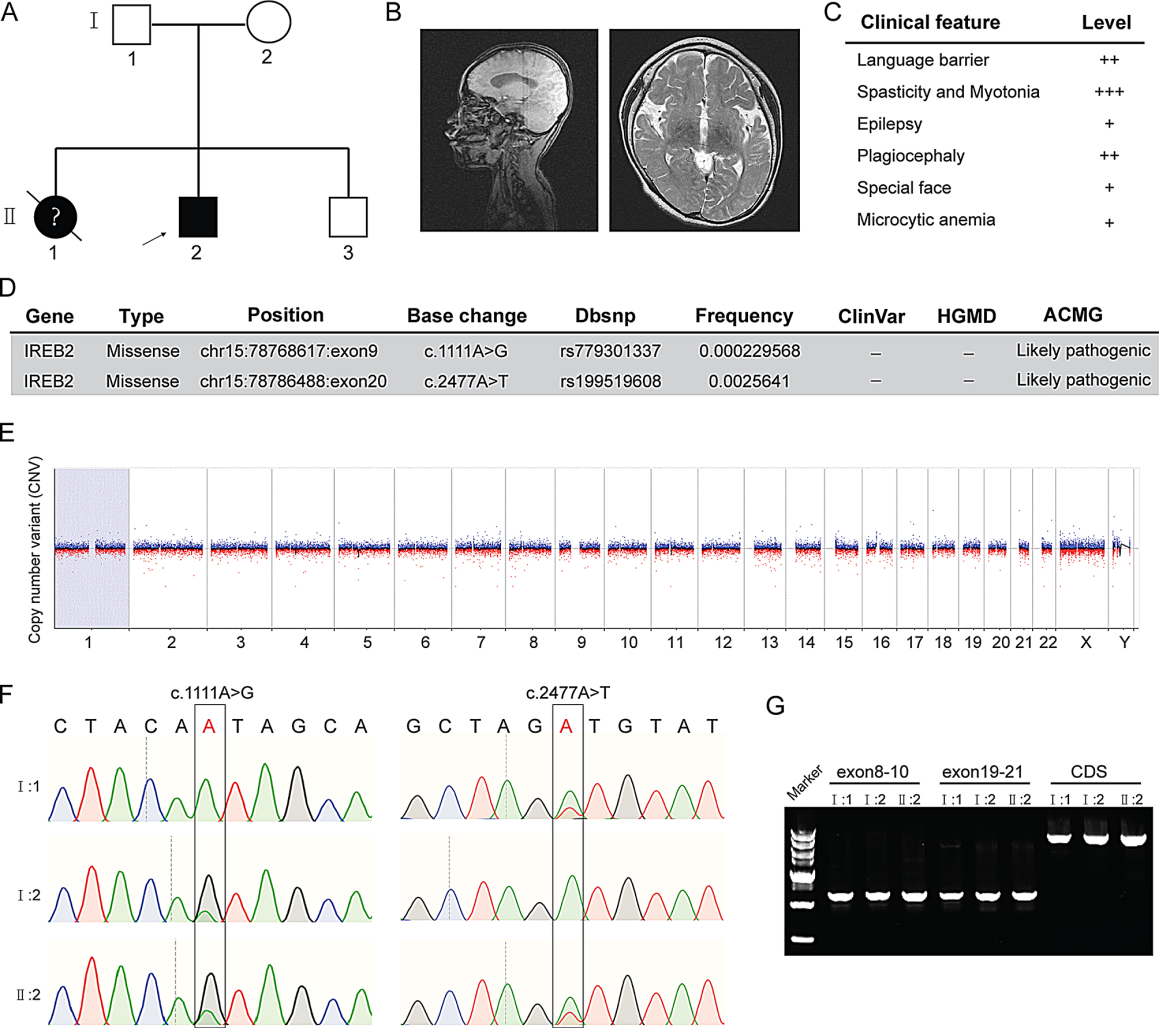
Genetic analysis of *IREB2* variants in a Chinese family due to neurodevelopmental delay in the patient. The arrow indicates the patient. **A**. Pedigree with the *IREB2* mutations in this study. **B**. Representative brain magnetic resonance images (MRI) of the patient. **C**. Clinical phenotypes and severity observed in the patient. **D**. Whole-exome sequencing (WES) analysis identified two compound heterozygous missense variants in the *IREB2* gene. **E**. Normal Copy Number Variation Sequencing (CNV-seq) result of the patient. **F**. Sanger sequencing confirmed the biallelic *IREB2* variants in the patient (II:2) inherited from his father (I:1, c.2477 A > T) and mother (I:2, c.1111 A > G). **G**. PCR products of the Exon regions containing mutation sites and the coding sequence (CDS) of *IREB2* gene in this family

The patient is an 8-year-old boy born to healthy, non-consanguineous Han Chinese parents following a normal pregnancy and full-term delivery (Fig. 1A). The pregnancy was complicated by turbid amniotic fluid. At birth, he was diagnosed with neonatal pneumonia and epilepsy, receiving 17 days of treatment in the Neonatal Intensive Care Unit (NICU). The patient presented with early global developmental delay, which was characterized by an inability to speak or walk, alongside severe dystonia, choreoathetoid movements, epilepsy, and non-specific facial dysmorphisms such as midface hypoplasia, short philtrum, low-set ears, and thick, wiry hair (Fig. 1C). His older sister also had developmental delays and feeding difficulties. Diagnosed with infantile spasms at around 4 months of age, she unfortunately passed away from renal failure at 6 months. The boy’s younger brother, born in 2023, is currently developing normally. A brain MRI at 8 years old revealed abnormal signals around the lateral ventricles, basal ganglia, and thalamus. It also showed a smaller and thickened corpus callosum, as well as bilateral frontotemporal subarachnoid spaces and deepened sulci, indicating loss of white matter and cerebral hypoplasia (Fig. 1B). Laboratory studies showed mild microcytic anemia, characterized by a hemoglobin level of 114 g/L (normal range: 120–140 g/L) and a mean corpuscular volume of 79.8 fL (normal range: 82–100 fL). Serum ferritin was measured at 24.34 ng/mL (normal range: 21.8–274.66 ng/mL), and iron levels were 7.84 µmol/L (normal range: 7.36–9.34 µmol/L).

### Identification and functional analysis of *IREB2* mutations in the NDCAMA patient

**Fig. 2:**
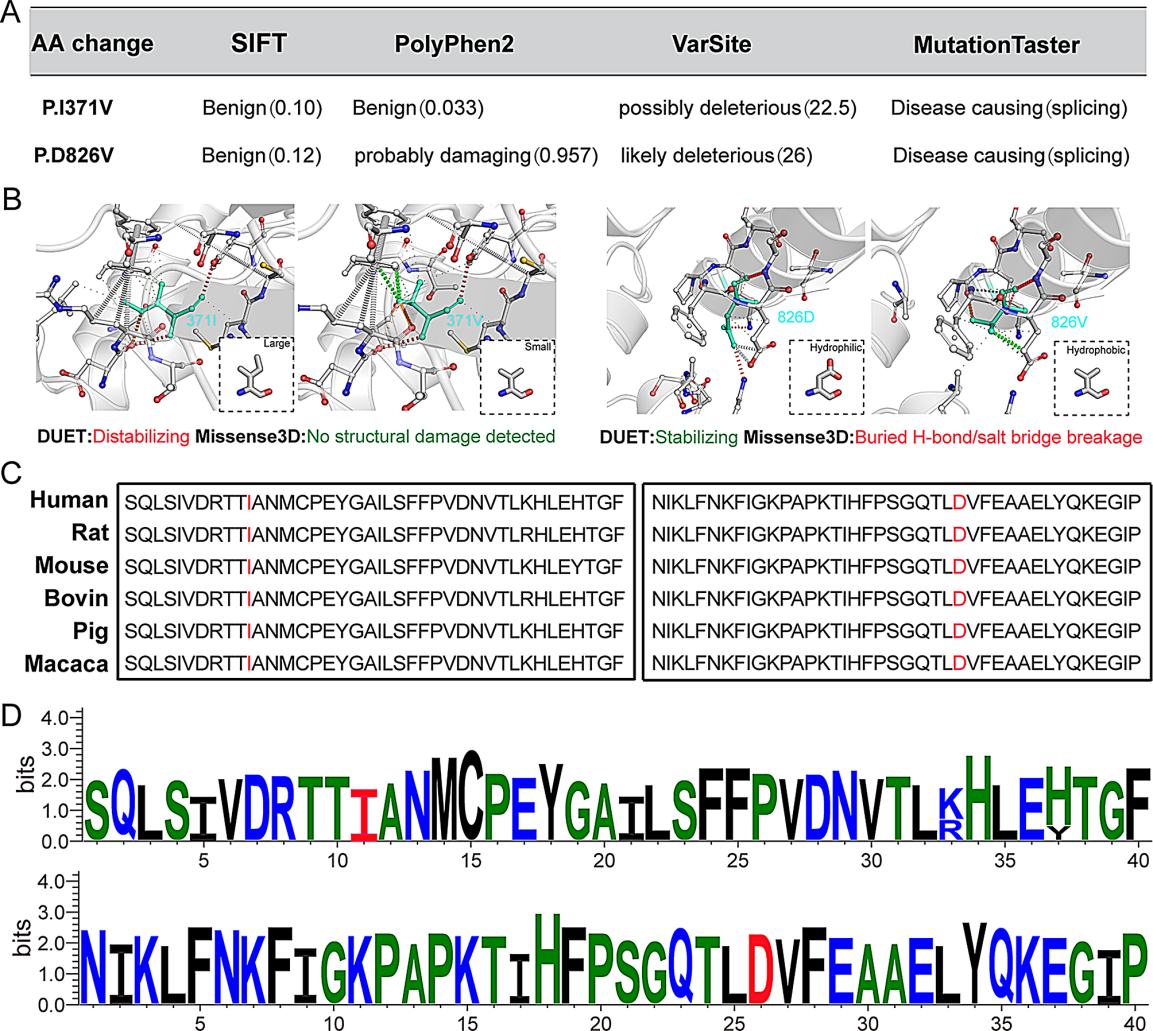
In silico predictions (**A**-**B**) and evolutionary conservation (**C**-**D**) of P.I371V and P.D826V on IRP2 protein

Clinical whole-exome sequencing (WES) identified compound heterozygous missense mutations in the NDCAMA*-*associated *IREB2* gene (GenBank: NM_004136.2; c.1111 A > G and c.2477 A > T). These mutations were classified as likely pathogenic according to American College of Medical Genetics and Genomics (ACMG) recommendations (Fig. 1D). Located in exons 9 and 20, these variants have a low population frequency and are not currently included in the HGMD and ClinVar databases. Copy number variant sequencing and inherited metabolic disease screening yielded negative results (Fig. 1E). Sanger sequencing confirmed the paternal (A2477T) and maternal (A1111G) origins of the variants, with PCR amplification of the sequence containing mutated sites and full-length coding sequencing of *IREB2* showing no splicing abnormalities (Fig. 1F-G). Both mutations, at positions Ile371 and Asp826, resulted in valine substitutions, which in silico analysis suggests deleterious effects (Fig. 2A). Specifically, P.Ile371Val may impact protein stability, while P.Asp826Val could lead to hydrophobicity alteration and structural change in IRP2 (Fig. 2B). Furthermore, the amino acid sequences at the mutation sites are highly conserved among different mammalian species (Fig. 2C-D). Unfortunately, the patient’s older sister did not undergo genetic testing, while his little brother carried the single A2477T mutation inherited from his father.

**Fig. 3:**
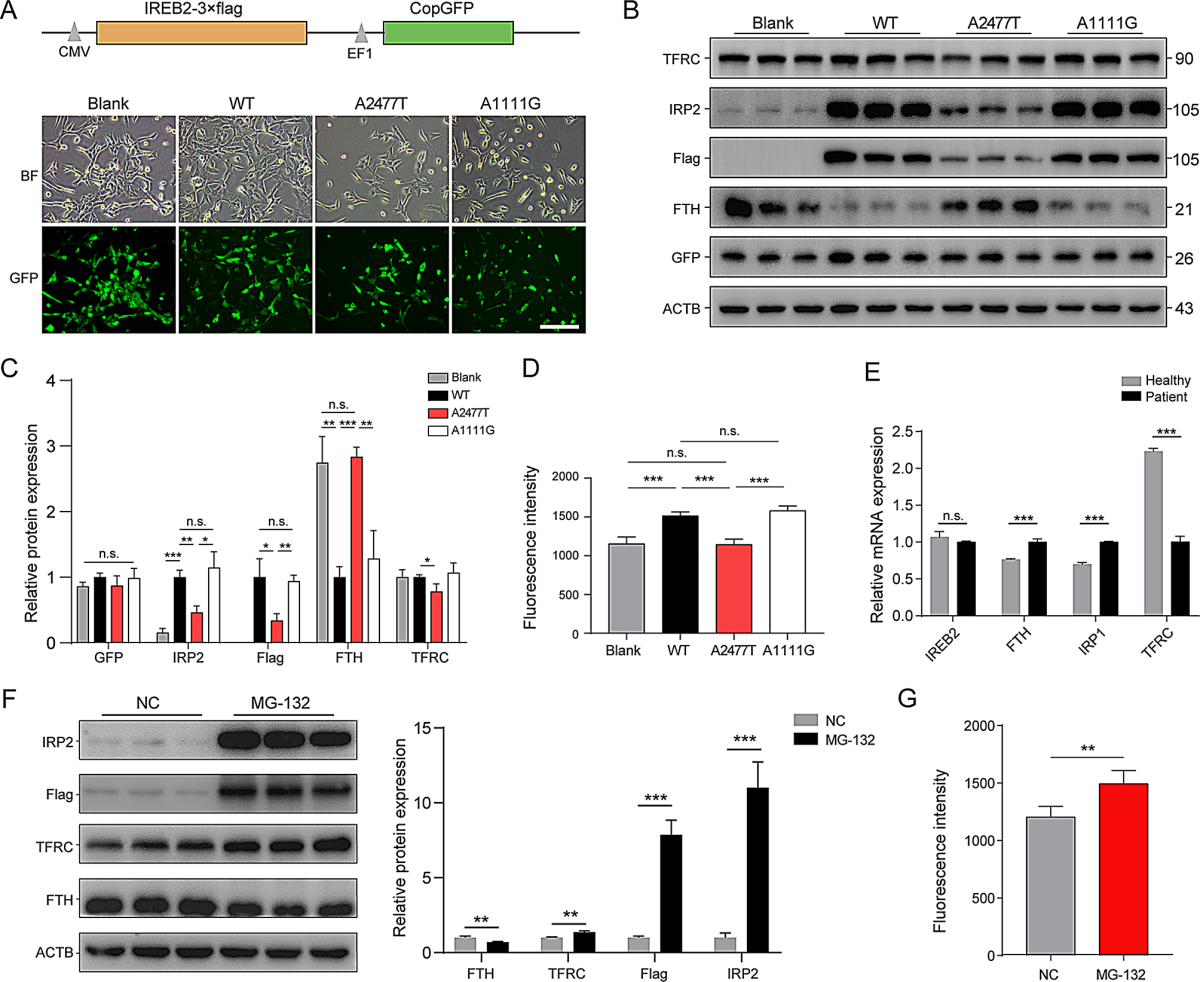
The impact of biallelic *IREB2* mutations on iron metabolism. **A**. SH-SY5Y cells were infected with lentivirus carrying Flag-tagged IREB2-wide-type (WT), A1111G, A2477T or empty vector (Blank), Scare bar = 100 μm. **B**. Expression of IREB2-regulated iron metabolism genes were detected by Western blot. **C**. Quantification of the Western blot results (*n* = 3, *p < 0.05, **p < 0.01, ***p < 0.001). **D**. Intracellular iron in different group was detected by the utilization of a FerroOrange fluorescent probe. **E**. RT-qPCR was performed to quantify mRNA levels of iron metabolism genes in healthy individual and the patient (*n* = 5, ***p < 0.001). **F**. Quantification of the western blot results to detect the protein levels of IRP2 and iron metabolism-associated proteins in A2477T cells treated with 40µM MG-132 or DMSO (*n* = 3, **p < 0.01, ***p < 0.001). **G**. Fluorescence intensity of intracellular iron levels in MG-132 treated cells or NC group (*n* = 5, **p < 0.01)

To further evaluate the impact of these biallelic variants on cellular iron metabolism, we used lentiviral infection to establish wild-type and mutant flag-tagged IRP2-overexpressing SH-SY5Y cells, a human neuroblastoma cell line. High infection efficiency was confirmed through GFP expression (Fig. 3A). Western blot analysis revealed that the A2477T mutation led to an approximate 70% reduction in IRP2 expression, as evidenced by the exogenously transferred flag-IRP2 expression (Fig. 3B). Compared to the wild-type group, the expression of FTH was notably increased while TFRC expression was significantly decreased. Conversely, the A1111G mutation did not impact the expression of iron metabolism proteins, although there was a slight increase in FTH expression (Fig. 3B-C). The intracellular Fe^2+^ level in the A2477T group was drastically reduced, similar to the control group, while no significant change was observed in the A1111G group. Furthermore, we examined the expression of *IREB2* and iron metabolism-related genes in patient-derived PBMCs. The results aligned with the A2477T mutation, showing indistinctive alteration in *IREB2* at the mRNA level, thus ruling out *IREB2* splicing abnormalities proposed in previously reported patients (Fig. 3E). The degradation of IRP2 protein plays an important role in regulating intracellular iron metabolism homeostasis [23]. Treatment with the proteasome inhibitor MG-132 led to IRP2 restoration and iron metabolism-related proteins’ expression, resulting in a remarkable increase in Fe^2+^ concentration (Fig. 3F-G). The findings suggest that the A1111G mutation is relatively mild, while the degradation of the IRP2 protein due to the A2477T mutation may play a key role in the pathogenesis of NDCAMA in this patient.

### Summary of reported cases and functional analysis of IREB2 mutations in NDCAMA

**Fig. 4:**
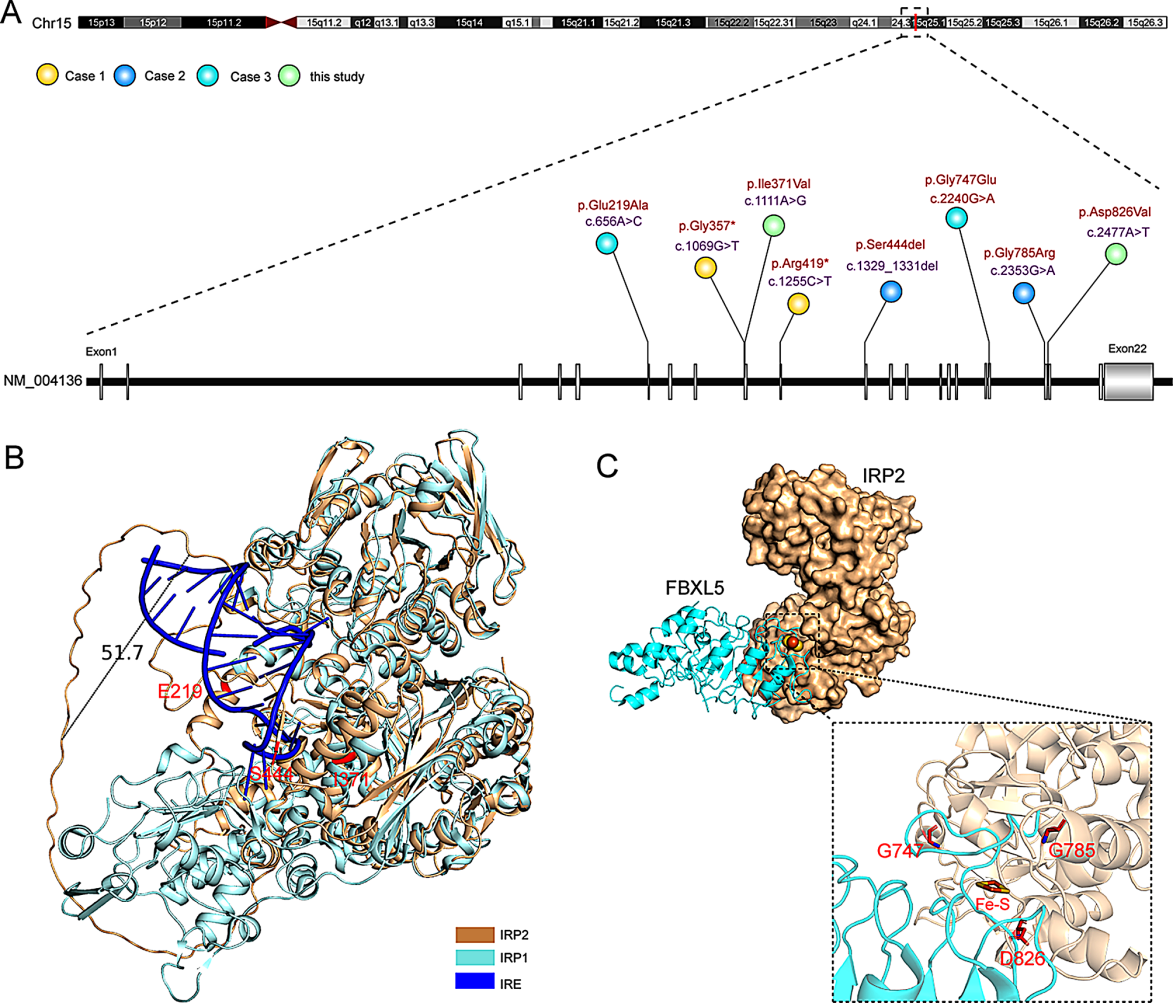
Known pathogenic variants in patients diagnosed with NDCAMA. **A**. Detailed view of the distribution of pathogenic IREB2 variants (GenBank: NM_004136.4) in 4 reported cases with base changes in purple and amino acid changes in red. Two representative views of the crystal structure including IRP2 binding to iron-responsive element (IRE) of ferritin (**B**), or FBXL5 (**C**) based on 6VCD and 3SNP. The amino acid residues mutated were depicted in red

A literature review has identified four reported cases of *IREB2*-associated NDCAMA (Fig. 4A, Table S2). Costain et al. (2019) reported the first case of a 16-year-old boy born to unrelated Filipino parents [18]. The second case, described by Cooper et al. (2019), involved a 10-year-old boy born to unrelated Australian parents [19]. Lastly, Maio et al. (2022) presented the third case of a 7-year-old boy born to healthy, non-consanguineous parents of Sephardic and Sephardic/Irish descent in the USA [20]. All three previously reported cases, along with our patient, exhibited similar clinical features, including neonatal feeding difficulties, hypotonia, choreoathetoid movements, impaired ambulation and communication, and non-specific facial dysmorphisms. Furthermore, brain imaging studies revealed progressive cerebral volume loss, delayed myelination, and a reduction in white matter volume. Notably, electroencephalograms (EEG) were abnormal in all three patients, although clinical seizures were not observed in the second patient described by Cooper et al. Additionally, laboratory studies indicated mild microcytic anemia, while serum iron levels remained within the normal range.

Whole-exome sequencing, or parallel gene sequencing, revealed that the patients were compound heterozygous for two variants in *IREB2*. The first case exhibited two nonsense mutations (c.1069G > T/p.Gly357* and c.1255 C > T/p.Arg419*), while the second case presented a missense mutation (c.2353G > A/p.Gly785Arg) accompanied by a 3-base pair in-frame deletion (c.1329_1331del/p.Ser444del). The third case, along with our patient, displayed two missense mutations (c.656 A > C/p.Glu219Ala and c.2240G > A/p.Gly747Glu). Cellular studies utilizing patient-derived lymphoblasts demonstrated a complete loss of IRP2 expression in the first case, resulting in altered post-transcriptional regulation of iron metabolism genes including *FTH* and *TFRC*, accompanied by a significant reduction in iron levels, which was primarily attributed to the mRNA surveillance pathway known as nonsense-mediated mRNA decay (NMD). Although cellular studies were not performed for the second case, in silico analyses indicated the potentially deleterious effects of the two mutations on iron-responsive element (IRE)-binding activity. In contrast, a dramatic decrease in IRP2 protein levels was observed in the patient-derived cells from the third case, where the patient also exhibited severe clinical symptoms. In the discussion, the authors proposed that mis-splicing of IREB2 mRNA and altered IRE-binding activities may elucidate the pathogenesis observed in the third patient. While these three cases were investigated directly using patient-derived cells, there remains a notable absence of experimental evidence to assess the detrimental effects of each mutation.

Unlike previous studies that examined compound heterozygous mutations, this study investigated the functions of the two mutations separately and identified c.2477 A > T mutation as deleterious, resulting in the degradation of IRP2. In contrast, the c.1111 A > G mutation appears to primarily affect iron-responsive element (IRE)-binding activity, which we classify as a relatively mild mutation.

Through modular docking and structure-based prediction, we identified the distribution pattern of compound heterozygous variants in NDCAMA patients. One mild mutation was located close to functional domains where IRP2 binds to the IRE element of iron metabolism-related mRNA, while the severe one was near the region where IRP2 binds to FBXL5, a key protein involved in IRP2 degradation (Fig. 4B). These findings may explain how compound heterozygous missense mutations contribute to abnormal iron metabolism in DNCAMA patients.

## Discussion

Our study expands the understanding of Neurodegeneration, Early-onset, with Choreoathetoid Movements and Microcytic Anemia (NDCAMA) by reporting a further case involving biallelic missense variants in the *IREB2* gene. This condition underscores the critical role of IRP2 in maintaining iron metabolism and mitochondrial function, with disruptions leading to severe neurological and hematological manifestations. The patient’s clinical presentations, including developmental delay, epilepsy, hypertonia, and microcytic anemia, aligns with previously reported NDCAMA cases. Notably, our patient exhibited novel compound heterozygous missense mutations (c.1111 A > G and c.2477 A > T) in *IREB2*, which were classified as likely pathogenic. These mutations, resulting in amino acid substitutions (Ile371Val and Asp826Val), are highly conserved across mammalian species and predicted to have deleterious effects on protein stability and function.

Functional analyses using patient-derived cells and SH-SY5Y cell lines overexpressing mutant IRP2 revealed significant degradation of the IRP2 protein in the presence of the A2477T mutation. This degradation was associated with altered expression of iron metabolism-related proteins, notably increased FTH and decreased TFRC, leading to reduced intracellular Fe^2+^ levels. In contrast, the A1111G mutation had a milder impact on these parameters. The findings from molecular docking and structural predictions suggest that these missense mutations disrupt critical functional domains of IRP2, particularly those involved in binding to iron-responsive elements (IRE) and the FBXL5 protein, which mediates IRP2 degradation. This disruption likely contributes to the observed abnormalities in iron metabolism and the pathogenesis of NDCAMA.

Previous studies have highlighted the role of IRP2 in regulating the metabolic switch from glycolysis to oxidative phosphorylation by stabilizing hypoxia-inducible factors. Our results further support the hypothesis that IRP2 degradation plays a pivotal role in the disease mechanism, as evidenced by the restoration of IRP2 expression and iron metabolism homeostasis upon treatment with the proteasome inhibitor MG-132.

## Conclusion

Our study identifies a further case of NDCAMA and elucidates the pathogenic mechanisms underlying IRP2-related neurodegeneration. The distinct effects of the A1111G and A2477T mutations on IRP2 stability and function provide valuable insights into the molecular basis of this rare disorder. Further research is needed to explore therapeutic strategies aimed at stabilizing IRP2 and correcting iron metabolism abnormalities in NDCAMA patients.

## Electronic supplementary material

Below is the link to the electronic supplementary material.

Supplementary Material 1

Supplementary Material 2

## Data Availability

All data supporting the findings of this study are available within the paper and its supplementary information files.

## Abbreviations

NDCAMA: Neurodegeneration, Early-onset, with Choreoathetoid Movements and Microcytix Anemia
WES: Whole Exome Sequencing
IREB2: Iron Responsive Element Binding Protein 2
FTH: Ferritin Heavy Chain
TFRC: Transferrin Receptor
FBXL5: F-Box and Leucine rich repeat protein 5

## References

[1] Andrews NC, Schmidt PJ. Iron homeostasis. Annu Rev Physiol. 2007;69:69–85. 17014365 10.1146/annurev.physiol.69.031905.164337

[2] Dutt S, Hamza I, Bartnikas TB. Molecular mechanisms of Iron and Heme Metabolism. Annu Rev Nutr. 2022;42:311–35. 35508203 10.1146/annurev-nutr-062320-112625 PMC9398995

[3] Camaschella C. Iron-deficiency anemia. N Engl J Med. 2015;372(19):1832–43. 25946282 10.1056/NEJMra1401038

[4] Zeidan RS, Han SM, Leeuwenburgh C, Xiao R. Iron homeostasis and organismal aging. Ageing Res Rev. 2021;72:101510. 34767974 10.1016/j.arr.2021.101510 PMC8620744

[5] Fang X, Ardehali H, Min J, Wang F. The molecular and metabolic landscape of iron and ferroptosis in cardiovascular disease. Nat Rev Cardiol. 2023;20(1):7–23. 35788564 10.1038/s41569-022-00735-4 PMC9252571

[6] Sawicki KT, De Jesus A, Ardehali H. Iron Metabolism in Cardiovascular Disease: Physiology, mechanisms, and therapeutic targets. Circ Res. 2023;132(3):379–96. 36730380 10.1161/CIRCRESAHA.122.321667 PMC9907000

[7] Levi S, Ripamonti M, Moro AS, Cozzi A. Iron imbalance in neurodegeneration. Mol Psychiatry. 2024;29(4):1139–52. 38212377 10.1038/s41380-023-02399-z PMC11176077

[8] Rouault TA. Iron metabolism in the CNS: implications for neurodegenerative diseases. Nat Rev Neurosci. 2013;14(8):551–64. 23820773 10.1038/nrn3453

[9] Ward RJ, Zucca FA, Duyn JH, Crichton RR, Zecca L. The role of iron in brain ageing and neurodegenerative disorders. Lancet Neurol. 2014;13(10):1045–60. 25231526 10.1016/S1474-4422(14)70117-6 PMC5672917

[10] Rouault TA. The role of iron regulatory proteins in mammalian iron homeostasis and disease. Nat Chem Biol. 2006;2(8):406–14. 16850017 10.1038/nchembio807

[11] Sanchez M, Galy B, Schwanhaeusser B, Blake J, Bahr-Ivacevic T, Benes V, Selbach M, Muckenthaler MU, Hentze MW. Iron regulatory protein-1 and – 2: transcriptome-wide definition of binding mRNAs and shaping of the cellular proteome by iron regulatory proteins. Blood. 2011;118(22):e168–179. 21940823 10.1182/blood-2011-04-343541

[12] Moroishi T, Nishiyama M, Takeda Y, Iwai K, Nakayama KI. The FBXL5-IRP2 axis is integral to control of iron metabolism in vivo. Cell Metab. 2011;14(3):339–51. 21907140 10.1016/j.cmet.2011.07.011

[13] Salahudeen AA, Thompson JW, Ruiz JC, Ma HW, Kinch LN, Li Q, Grishin NV, Bruick RK. An E3 ligase possessing an iron-responsive hemerythrin domain is a regulator of iron homeostasis. Science. 2009;326(5953):722–6. 19762597 10.1126/science.1176326 PMC3582197

[14] Wang H, Shi H, Rajan M, Canarie ER, Hong S, Simoneschi D, Pagano M, Bush MF, Stoll S, Leibold EA, et al. FBXL5 regulates IRP2 Stability in Iron Homeostasis via an oxygen-responsive [2Fe2S] cluster. Mol Cell. 2020;78(1):31–e4135. 32126207 10.1016/j.molcel.2020.02.011 PMC7159994

[15] Meyron-Holtz EG, Ghosh MC, Iwai K, LaVaute T, Brazzolotto X, Berger UV, Land W, Ollivierre-Wilson H, Grinberg A, Love P, et al. Genetic ablations of iron regulatory proteins 1 and 2 reveal why iron regulatory protein 2 dominates iron homeostasis. EMBO J. 2004;23(2):386–95. 14726953 10.1038/sj.emboj.7600041 PMC1271751

[16] Ghosh MC, Tong WH, Zhang D, Ollivierre-Wilson H, Singh A, Krishna MC, Mitchell JB, Rouault TA. Tempol-mediated activation of latent iron regulatory protein activity prevents symptoms of neurodegenerative disease in IRP2 knockout mice. Proc Natl Acad Sci U S A. 2008;105(33):12028–33. 18685102 10.1073/pnas.0805361105 PMC2497459

[17] LaVaute T, Smith S, Cooperman S, Iwai K, Land W, Meyron-Holtz E, Drake SK, Miller G, Abu-Asab M, Tsokos M, et al. Targeted deletion of the gene encoding iron regulatory protein-2 causes misregulation of iron metabolism and neurodegenerative disease in mice. Nat Genet. 2001;27(2):209–14. 11175792 10.1038/84859

[18] Costain G, Ghosh MC, Maio N, Carnevale A, Si YC, Rouault TA, Yoon G. Absence of iron-responsive element-binding protein 2 causes a novel neurodegenerative syndrome. Brain. 2019;142(5):1195–202. 30915432 10.1093/brain/awz072 PMC6487337

[19] Cooper MS, Stark Z, Lunke S, Zhao T, Amor DJ. IREB2-associated neurodegeneration. Brain. 2019;142(8):e40. 31243445 10.1093/brain/awz183

[20] Maio N, Saneto RP, Steet R, Sotero de Menezes MA, Skinner C, Rouault TA. Disruption of cellular iron homeostasis by IREB2 missense variants causes severe neurodevelopmental delay, dystonia and seizures. Brain Commun. 2022;4(3):fcac102. 35602653 10.1093/braincomms/fcac102 PMC9118103

[21] Li H, Liu Y, Shang L, Cai J, Wu J, Zhang W, Pu X, Dong W, Qiao T, Li K. Iron regulatory protein 2 modulates the switch from aerobic glycolysis to oxidative phosphorylation in mouse embryonic fibroblasts. Proc Natl Acad Sci U S A. 2019;116(20):9871–6. 31040213 10.1073/pnas.1820051116 PMC6525483

[22] Guo Z, Geng M, Huang Y, Han G, Jing R, Lin C, Zhang X, Zhang M, Fan G, Wang F, et al. Upregulation of Wilms’ Tumor 1 in epicardial cells increases cardiac fibrosis in dystrophic mice. Cell Death Differ. 2022;29(10):1928–40. 35306537 10.1038/s41418-022-00979-0 PMC9525265

[23] Rouault TA, Maio N. How oxidation of a Unique Iron-Sulfur Cluster in FBXL5 regulates IRP2 levels and promotes regulation of Iron Metabolism proteins. Mol Cell. 2020;78(1):1–3. 32243827 10.1016/j.molcel.2020.03.020 PMC10113915

