# Supplementary material for: Novel biallelic variants in IREB2 cause an early-onset neurodegenerative disorder in a Chinese pedigree
Source: Orphanet J Rare Dis. 2024 Nov 25;19:435. doi: 10.1186/s13023-024-03465-7 (PMC11587613; doi:10.1186/s13023-024-03465-7)
Supplement: Supplementary file 1 — Supplementary Material 1 [file 13023_2024_3465_MOESM1_ESM.docx]

Table S2. Literature review of reported NDCAMA patients including this study.

| **Cases** | **Patient** | **Base change** | **AA chanege** | **Mutation Type** | **Comment on Severity** | **Molecular characteristics** | **Clinical features** |
| --- | --- | --- | --- | --- | --- | --- | --- |
| Costain et al.  (2019) | 16-year-old male | c.1069G>T | p.Gly357* | Nonsense | Severe | **a. Cell model**: Patient-derived lymphoblasts  **b. IRP2 Protein level**: Undetectable  **c. Altered regulation of iron metabolism genes**: YES  **d.** **Iron level**: Reduction  **e. Mechanism:** Nonsense-mediated mRNA decay | **Developmental delays**  1) Feeding problems  2) Poor head control, hypotonia, and dystonic posturing  3) Choreoathetoid movements  4) Absent speech  5) Clinical seizures |
|  | Filipino | c.1255C>T | p.Arg419* | Nonsense | Severe |  |  |
| Cooper et al.  (2019) | 10-year-old male | c.1329_1331del | p.Ser444del | Indel | Mild | **a-d.** None  **e. Mechanism:** IRE-binding activity | **Facial dysmorphisms**  1) Midface hypoplasia  2) Exotropia  3) Short philtrum, low-set ears |
|  | Australian | c.2353G>A | p.Gly785Arg | Missense | Severe |  |  |
| Maio  et al.  (2022) | 7-year-old  male | c.656A>C | p.Glu219Ala | Missense | Mild | **a. Cell model**: Patient-derived lymphoblasts  **b. IRP2 Protein level**: Decreased  **c. Altered regulation of iron metabolism genes**: YES  **d.** **Iron level**: Reduction  **e. Mechanism:** Mis-splicing and IRE-binding activity | **Neuroimaging findings**   1. Cerebral atrophy with white matter loss   2) Delayed myelin maturation  3) Abnormal EEG |
|  | Sephardic and Sephardic/Irish in USA | c.2240G>A | p.Gly747Glu | Missense | Severe |  |  |
| This study  (2024) | 8-year-old  male | c.1111A>G | p.Ile371Val | Missense | Mild | **a. Cell model**: Patient-derived PBMCs and SH-SY5Y cell line  **b. IRP2 Protein level**: Decreased  **c. Altered regulation of iron metabolism genes**: YES  **d.** **Iron level**: Reduction  **e. Mechanism:** Protein degradation and IRE-binding activity | **Laboratory studies**  Microcytic anemia with normal serum Iron levels |
|  | Chinese | c.2477A>T | p.Asp826Val | Missense | Severe |  |  |
